# The Effects of Construction and Demolition Waste (C&DW) Fine Residues on Landfill Environments: A Column Leaching Experiment

**DOI:** 10.3390/toxics13050370

**Published:** 2025-05-02

**Authors:** Adane S. Molla, Waiching Tang, Willy Sher, Md Mezbaul Bahar, Dawit Nega Bekele

**Affiliations:** 1 School of Architecture and Built Environment, University of Newcastle, University Dr, Callaghan, NSW 2308, Australia; 2 Global Centre for Environmental Remediation, University of Newcastle, Callaghan, NSW 2308, Australia; mezbaul.bahar@newcastle.edu.au (M.M.B.); dawit.bekele@newcastle.edu.au (D.N.B.); 3 CRC for Contamination Assessment and Remediation of the Environment (crcCARE), Callaghan, NSW 2308, Australia; 4 Douglas Partners Pty Ltd., Coorparoo, QLD 4151, Australia

**Keywords:** C&DW fine residues, heavy metal mobilization, gypsum-induced leaching, landfill ecotoxicity, sulfate-metal complexation, emerging contaminant, toxic leachate dynamics

## Abstract

The rapid increase in construction and demolition waste (C&DW) has emerged as a significant environmental challenge, particularly due to the hazardous substances embodied within the fine residues destined into landfills. The disposal of C&DW in landfills has been widely recognized as a source of leachate, containing toxic contaminants, which pose significant environmental risks. A controlled column leaching experiment was conducted using samples with varying proportions of C&DW, gypsum, and organic content to assess their impact on leachate chemistry. The results indicate that higher C&DW content leads to increased concentrations of heavy metals, such as Pb, Hg, As, Cr, Ni, Cu, Zn, and Co, as well as other metals like Al and Fe, with peak contamination occurring within the first 13–15 weeks. Gypsum presence exacerbates heavy metal solubility by reducing pH, increasing sulfate levels, and promoting metal-sulfate complex formation. Despite remaining within regulatory thresholds, the cumulative concentration of toxic metals over time highlights potential environmental risks, particularly in landfill settings. This study underscores the need for improved C&DW management practices, enhanced waste segregation, and sustainable alternatives to gypsum to mitigate long-term ecological impacts. These findings contribute to a deeper understanding of C&DW leachate dynamics and inform policy recommendations for sustainable waste management in the construction sector.

## 1. Introduction

The sevenfold increase in the world population over the past two centuries has greatly amplified humanity’s impact on the natural environment. According to United Nations projections, the global population, currently at 8.12 billion, is expected to rise to 10.4 billion people by the end of the century [1,2]. This increase will be a significant factor for industrial development. It will necessitate an increase in construction worldwide to accommodate the larger population. On the other hand, it is widely known that construction activities consume a large amount of resources and natural materials but also generate substantial waste [3,4]. This poses a major threat to the environment and ecosystems [5,6,7].

The Australian National Waste Report reveals that construction and demolition waste (C&DW) was the largest contributor to overall core waste in 2018–2019, making up nearly 44% of the total waste generated. Of this, 23.2% was sent to landfills [8]. Additionally, data from the past 13 years show a 61% increase in C&DW, with most of this growth occurring in the last five years, coinciding with a rapid phase of urban development [9]. Managing this growing volume of C&DW presents a significant global challenge as a substantial portion ends up in landfills, leading to the production of harmful leachates [10,11,12,13]. Added to this complexity, recent anti-waste movements by countries such as China, Thailand, Philippines, and Malaysia that ban the import of recyclables from developed nations have left Australia with worrying rates of stockpiled waste [14,15,16,17,18,19,20]. This had forced Australian material recovery facilities (MRFs) to resort to source-segregated recyclables meaning that several local councils have had to find places to dump mixed waste streams including C&DW in landfills [17,18].

Mixed C&DW can contain hazardous materials, the mixture of which can cause risks to both the environment and human health [21,22]. To mitigate the environmental and human health risks associated with the management and/or disposal of C&DW, it is crucial to understand how hazardous materials from mixed C&DW interact with the environment. Several approaches can be employed to study this interaction, including investigating how pollutants leach from disposal or containment structures, analyzing the composition of spent materials or leachates, conducting environmental inventories, and undertaking long-term monitoring. Various researchers have investigated the leaching characteristics of C&DW materials to assess their potential for reuse. Delay et al. [23] conducted column and lysimeter leaching tests to measure the release of inorganic pollutants in recycled C&DW. Similarly, Yong-Chul and Timothy [24] employed batch and column leaching procedures to analyze the leaching behavior of sulfate and dissolved solids from C&DW fine residues. Several other studies have also focused on characterizing hazardous leachates from C&DW mixtures. For instance, Roussat et al. [25] examined the release potential of CCA-treated wood and painted wood using lysimeter leaching tests. Furthermore, research by Jambeck et al. [26] and Khan et al. [27] evaluated the concentrations and leaching rates of arsenic, copper, and chromium in municipal solid waste (MSW) and C&DW lysimeter tests.

The composition of C&DW fine residues greatly affects the quality of leachate produced at the landfills, subsequently resulting in groundwater contamination. When organic materials mix with plasterboard (gypsum, calcium sulfate dehydrate, and CaSO_4_· 2H_2_O), landfill biological processes result in the generation of hydrogen sulfides (H_2_S) and sulfuric acid (H_2_SO_4_) [28]. This results in a change in the pH of the leachate resulting in reductive dissolutions. These can mobilize metals such as arsenic, copper, and chromium that are commonly found in treated wood fine residues within the waste stream and other toxic heavy metals from naturally occurring materials [28,29]. A groundwater monitoring study on unlined landfills that receive C&DW in Florida reported a down gradient high concentration of sulfate, iron, and arsenic, suggesting leachate contamination from C&DW [30].

An over-reliance on mechanized demolition technologies due to ever-increasing labor costs for selective deconstruction has resulted in the generation of a higher proportion of mixed C&DW. This huge volume of mixed C&DW needs to be sorted to feed recovery processes in MRFs. This involves a series of shredding and size reduction processes and subsequent mixing. In turn, this generates significant quantities of potentially hazardous contaminants as C&DW fine residues. Given the current management of residuals and their disposal practices [31], the impact of C&DW fine residues disposal in Australia has not been adequately investigated [22].

The aim of this study is to the following:

i. Assess the levels and release potential of heavy metals from C&DW fine residues.
ii. Investigate the impact of C&DW and gypsum content on the leaching and release behavior of heavy metals.
iii. Determine environmentally friendly C&DW–gypsum content and identify combinations that pose environmental risks.

The study will enhance understanding of the environmental risks linked to the disposal of C&DW fine residues, which are typically considered inert. It will also help identify the potential destiny of contaminants in landfills and evaluate the effectiveness of containment measures in protecting the environment and human health. By identifying the levels of hazardous substances within C&DW fines, the study will provide valuable insights that can enhance waste management practices, inform policy and regulatory development, and expand the knowledge base necessary for more efficient recycling and resource recovery methods. This research supports a circular economy and is globally relevant, addressing challenges posed by international bans on recyclable imports and contributing to the scientific community’s understanding of C&DW management and its environmental impacts.

## 2. Materials and Methods

### 2.1. Study Area and Site Selection

**Figure 1:**
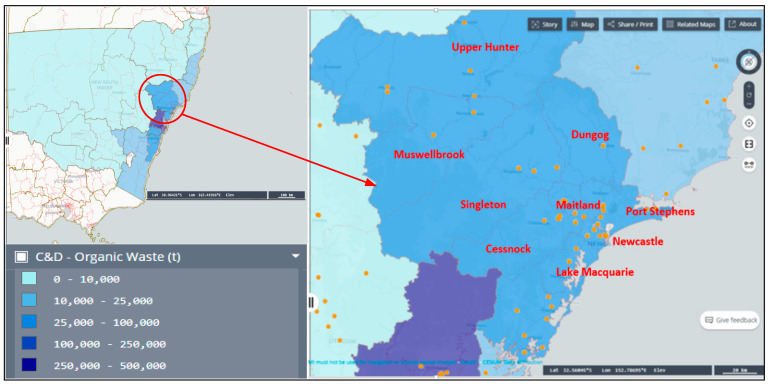
Map of Hunter/Central Coast region, NSW, Australia.

According to data obtained from the New South Wales (NSW) public registers [32], there are 210 facilities in the NSW region licensed and operating for handling C&DW under the license “Waste storage–other types of waste”. Moreover, data provided by the Australian Biomass for Bioenergy Assessment (ABBA) project [33] have stratified the whole of NSW based on tonnes of C&DW received at waste transfer facilities within the state. As depicted in Figure 1, councils within the Hunter/Central Coast region receive a huge amount of C&DW. Moreover, this sub-region is a region of concern based on NSW Environmental Protection Authority (EPA)’s “Waste Less, Recycle More initiative” regarding plasterboard recycling [34]. The Hunter/Central Coast region was thus selected as the focus area of this study, providing three benefits. Firstly, it represents regions that receive the highest amount of C&DW as well as those that receive the least. Secondly, prevailing conservative plasterboard recovery practices offer additional opportunities to investigate the impact of the particular project. Thirdly, councils within this sub-region have established a common joint institution that specifically deals with waste management efforts.

Furthermore, data obtained from the Australian Renewable Energy Agency (ARENA) identify 45 C&DW recovery facilities in this sub-region [35]. Although detailed information on the type of recovery facilities is limited, various reports indicate that fixed C&DW processing and recovery facilities—permanent sites where construction and demolition waste is sorted, processed, and recycled into reusable materials—are scarce even at the state level. Hence, all fixed C&DW sorting and processing plants within this sub-region were included in this study. However, several consultations with EPA, NSW experts, and local government officials as well as industry practitioners identified only one facility that undertook mixed C&DW sorting and processing in this region. The rest of the facilities principally operated as transfer stations to source separated C&DW, and no processing was conducted in these sites.

### 2.2. Experimental Setting

**Figure 2:**
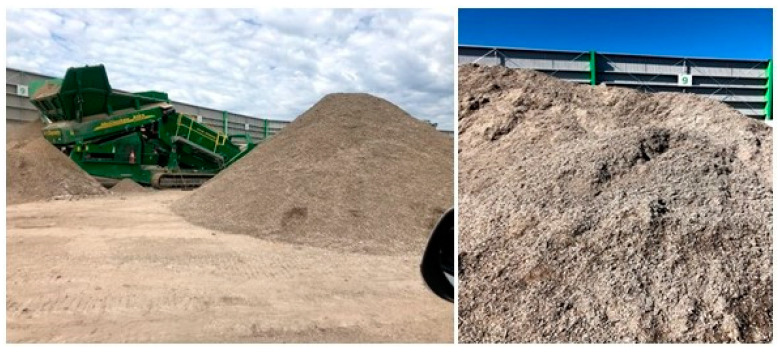
C&DW processing residual fines accumulated for disposal into landfills.

A hypothetical representation of common landfills operating in and around the Hunter/Central coast sub-region was simulated in a laboratory column experiment conducted at the University of Newcastle, Newcastle Institute for Energy and Resources (NIER) facility. Each column was packed with organic matter, C&DW fine residues, and gypsum. C&DW fine residue samples were collected from multiple stockpiles within an MRF (Figure 2). Proportionally allocated sub-samples were acquired from each stockpile. Depending on the pile size, a reduction in sample size was achieved by successive coning and quartering method [36,37,38,39], and a final sample size of 20 L was collected from each pile. The different C&DW fine residue batch samples were then transported to the University of Newcastle soil shed and mixed in a large cement mixer to get a uniformly mixed C&DW fine residue Waste construction gypsum board was crushed and dried in an oven at a temperature of 60 °C for 24 h [40]. Once the gypsum particles were dried, a portion was crushed to 2 mm size using a pestle and mortar. Commercially available organic compost was used to represent the organic fraction of a simulated landfill. After preparing the column setup, sampling and analysis of leachates were conducted fortnightly with continuous feeding to maintain the column saturated throughout the experiment.

### 2.3. Column Setup and Material of Construction

**Figure 3:**
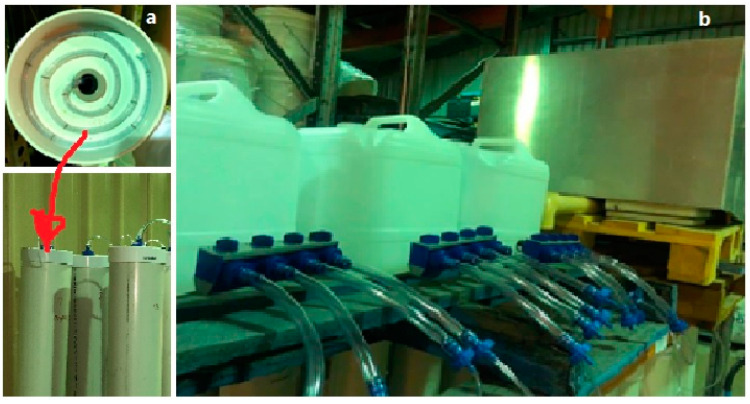
Column top cover with coiled flow distribution vinyl pipe (**a**) and feed tank arrangements (**b**).

A storm water UPVC (Unplasticized Polyvinyl Chloride) pipe of 150 mm diameter was used to construct each column. The same material was used for bottom and top caps. The top cap was perforated at the center to provide an inlet for feeding column leaching fluid and the bottom outlet for the leachate. The top was fitted with three circles of perforated 5 mm clear vinyl pipe to feed leaching fluid as shown in Figure 3a. The three perforated coiled vinyl pipes were intended to distribute the leaching fluid simulating rain distribution and prevent preferential flow of leaching fluid. On the other hand, the collection of leachates at the bottom outlet was via a 13 mm clear vinyl pipe fitted with end plug. A 15 L High-Density Polyethylene tank was fitted to a flow splitter to 4 individual columns (Figure 3b). Sampling ports were also fitted with a plug to increase retention and contact time of leaching fluid to column contents. The feeding tanks were fitted with a flow distribution tap with adjustable flow control valve.

### 2.4. Leaching Column Composition and Packing

Eighteen individual columns were prepared, each packed with organic matter (local organic compost that can represent the organic fraction of landfill waste) and C&DW with or without gypsum to a height of 75 cm. The packed column mix was prepared with quantities of mixture proportions described in Table 1.

**Figure 4:**
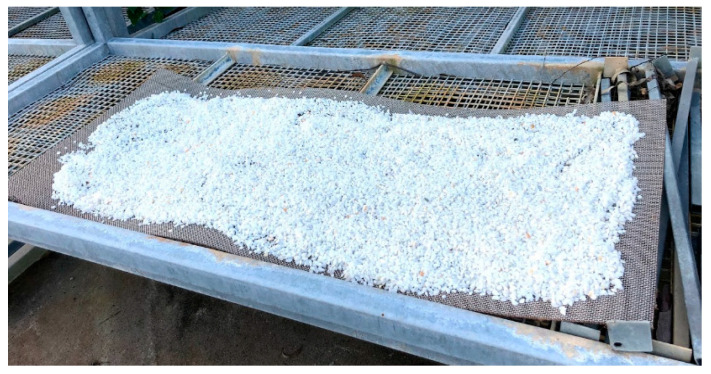
Draining of washed pebbles for 6 hrs before putting them inside an oven.

Each column was bedded with fine plastic mesh to prevent soil particles from blocking the leachate outlet. A 4 cm layer of quartz pebbles (2–6 mm) was then placed on top of the mesh, followed by a 4 cm layer of washed coarse sand. Both the pebbles and coarse sand were washed three times, spread over a mesh to drain the water for 6 h, and then oven-dried at 60 °C for 48 h before being packed (Figure 4)

**Figure 5:**
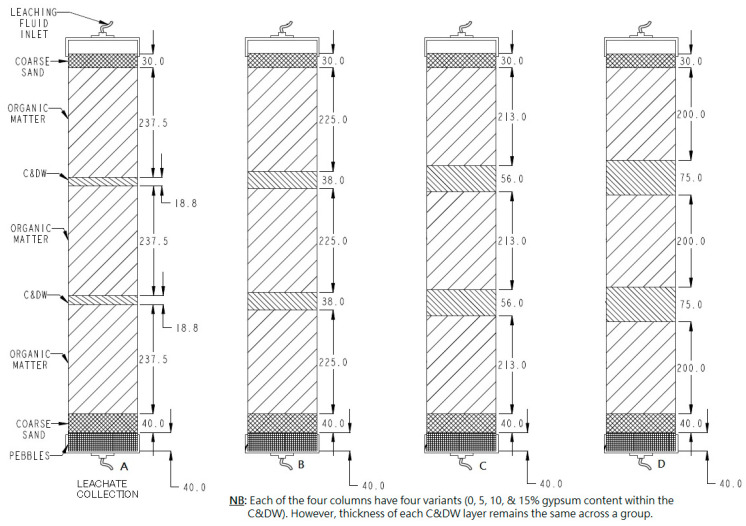
Column mix proportions. A, B, C, and D represent column mixes of 5, 10, 15, and 20% C&DW each with 0, 5, 10, and 15% gypsum content variants as specified in Table 1 above.

**Figure 6:**
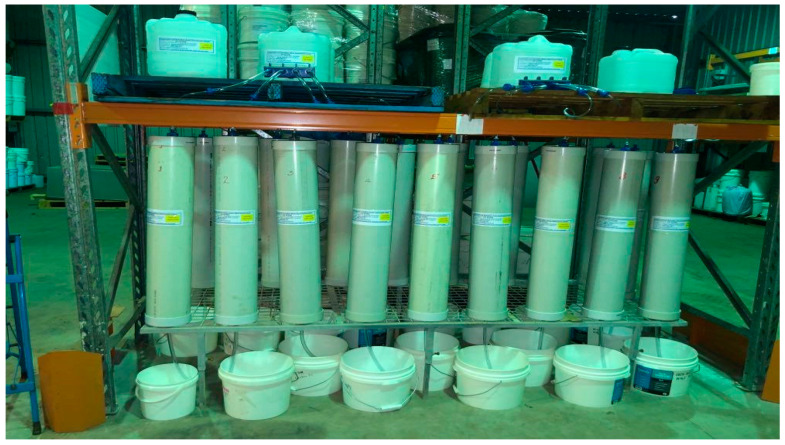
Complete setup of leaching apparatus and leachate collection arrangements.

The C&DW mixed with gypsum was placed at three equal intervals (every 20–23.75 cm) as shown in Figure 5. Each of the three layers of the weighed organic portion was divided into two equal parts (totally 6 portions per column) to facilitate column filling uniformity and avoid differential settling. During filling, every portion was tapped round 8 times on the outside surface of the column using a trowel handle to prevent void spaces that might cause preferential flow of leaching fluid. The full column setup is shown in Figure 5 and Figure 6.

### 2.5. Leaching Fluid Preparation, Feeding, and Sampling

Tap water was connected directly to the feeding tanks with a hose (5 feeding tanks of 15 L capacity). In total, 450 µL of 60/40 *w*/*w*% H_2_SO_4_/HNO_3_ was added to each of the feeding tanks to alter the pH of the leaching fluid to that of local precipitation (pH = 4.5) [41,42]. The columns were fed with the leaching fluid until they were fully saturated at a flow rate of 10–15 drops/min (the minimum practicable flow rate that could be achieved) with the bottom outlet remaining closed until sampling. Considering the average annual rainfall in the Hunter/Central Coast subregion (870 mm/year) [43] and translating it into the cross-section of the column, the potential flow rate was calculated at 5 drops/min. However, achieving a flow rate of 5 drops/min with this setup proved impractical. The minimum achievable flow rate was 10–15 drops/min. Attempts to reach this flow rate through manual adjustment were time-intensive and ultimately not feasible. Consequently, as this arrangement resulted in a doubling or tripling of the intended feed rate of 5 drops/day, feeding was limited to specific intervals rather than a continuous 24 h period. The system was instead operated for an average of 8 h daily at the specified flow rate.

After full saturation, the bottom outlets of leachate were closed to enable sufficient contact time with the leaching fluid. After one week, baseline sampling been conducted. During sampling, some of the bottom leachate not in direct contact with the column contents was drained for approximately 10 min and discarded. This ensured that representative samples of the column contents were obtained. After first sampling, the leaching fluid was replaced to full saturation and outlets were recapped. Based on indications from week one, the next sampling was taken fortnightly.

### 2.6. Determination of Heavy Metals and Major Cations

#### 2.6.1. Sample Preparation

Ten metals were targeted for analysis (Aluminum, Chromium, Iron, Cobalt, Nickel, Copper, Zinc, Arsenic, Mercury, and Lead). Leachate samples were collected fortnightly using 50 mL centrifuge tubes and pH and conductivity were measured by a coupled pH-conductivity meter (METTLER TOLEDO International Inc., SevenCompact™ Duo S213, Greifensee, Switzerland). The samples were then centrifuged and 10 mL portion of it was filtered through 0.45 µm syringe filters and transferred into 10 mL tubes for analysis. Another 0.5 ml portion of the filtered sample was diluted 20 times and transferred into a 10 mL ICP tube for analysis of mercury and major cations. All chemical methods were administered according to the Australian Laboratory Handbook for soil and water chemical analyses [44]. All glassware was prewashed with copious amounts of water and then soaked for at least 6 h in 3% HNO_3_ solution. After soaking, the glassware was rinsed thoroughly with tap water, followed by a final rinse with deionized water before every use.

The authors confirm that safety clearance for the experimental procedures conducted in this study was obtained from the University of Newcastle Health and Safety Team.

#### 2.6.2. Apparatus

Inductively Coupled Plasma Mass Spectrometry (PerkinElmer Inc., NexION^®^ 350X ICP-MS, Waltham, MA, USA) was used for the analysis of heavy metals and Inductively Coupled Plasma Optical Emission Spectrometer (ICP-OES, PerkinElmer Avio 200 model) was used for analysis of cations such as (Al and Fe).

#### 2.6.3. Reagents and Chemicals

All reagents and chemicals used were high-purity analytical grade chemicals obtained from Thermo Fisher Scientific Inc., Waltham, MA, USA, and Sigma-Aldrich Pty Ltd., Castle Hill, NSW, Australia, an affiliate of Merck KGaA, Darmstadt, Germany. The main chemicals and reagents utilized were hydrochloric acid (HCl), 37% (ACS grade reagent); nitric acid (HNO_3_), 70%, d = 1.42 (ACS grade reagent); sulfuric acid (H_2_SO_4_), 98% SG 1.83, and Milli-Q water (18.2 MΏ).

#### 2.6.4. Analysis of Heavy Metals and Cations (ICP-MS and ICP-OES)

Elemental concentrations of Pb, Hg, As, Cr, Ni, Cu, Zn, and Co were determined using ICP-MS (PerkinElmer NexION 350X), while Al and Fe were analyzed using ICP-OES (PerkinElmer Inc., Avio^®^ 200 ICP-OES). For ICP-MS, internal calibration was prepared using a multi-element internal standard mixture containing Sc-45, Y-89, Rh-103, In-115, and Tb-159, each at a concentration of 10 ppm in a 2% HNO_3_ matrix. The PerkinElmer NexION 350X Series ICP-MS can measure trace elements as low as one part per trillion (ppt) and can quickly scan more than seventy elements to determine the composition of an unknown sample. The ICP-MS system is automated with Agilent Technologies Inc., MassHunter 4.3, Workstation Software, Santa Clara, CA, USA, which accurately interprets the resulting data. The instrument features an on-board peristaltic pump to control the flow of sample solution into and waste (drain) out of the instrument, a Micro Mist nebulizer that uses a stream of argon to disperse the sample, an ICP Argon plasma torch using Argon as plasma gas, auxiliary gas and nebulizer (carrier) gas, two pumps for evacuation, a quadrupole mass analyzer with 0.8 amu resolution at 10% height, an Octapole Reaction System (ORS), and electron multiplier detector. The operating conditions are as follows: nebulizer gas (Ar) flow rate: 0.9 L/min, auxiliary gas (Ar) flow 0.3 L/min, plasma (Ar) gas flow rate: 15 L/min, reaction gas flow (He) 4 mL/min. Lens voltage was maintained at 7.25 V, with an RF power setting of 1100 W. The oxide ratio (CeO^+^/Ce^+^) and doubly charged ion ratio (Ce^+2^/Ce^+1^) were both kept at 1% reflecting effective plasma performance and minimal polyatomic interference.

For ICP-OES, multi-element standard solutions (CertiPUR^®^ from Merck) containing known concentrations of elements including Ca, Mg, K, Na, Fe, Mn, and Al were prepared in a matrix of 1% HNO_3_ and diluted across a concentration range of 0.01 to 10 mg/L to construct calibration curves. The PerkinElmer Avio 200 model of ICP-OES features a dual-view configuration, enabling both axial and radial plasma observation to accommodate trace and matrix-rich samples. Key operating parameters for the ICP-OES analysis were RF power (1500 W), plasma gas (Ar) flow rate (10 L/min), auxiliary gas (Ar) flow rate (0.5 L/min), nebulizer gas (Ar) flow rate (0.7 L/min), and sample uptake rate (~1.0 mL/min)

To ensure accuracy and consistency across all measurements, parallel samples and replicate analysis were employed, and both calibration standards and samples were matrix matched and prepared in the same acid concentration. Each leachate sample was prepared by diluting 1.0 mL of the original sample to 10.0 mL with deionized water. All samples were analyzed in triplicate, and results were expressed as mean ± standard deviation (SD). The relative standard deviations (RSDs) of the triplicate measurements were calculated and found to be less than 5% for all elements analyzed, indicating high analytical precision. Furthermore, six-point calibration curves for all metals were constructed by plotting the ratio of the intensity of the analyte metal to that of the internal standard (IS) against the concentration of the trace metal. All calibration curves exhibited excellent linearity with a correlation coefficient (r^2^ > 0.999) for all metals analyzed. Internal standard recoveries were within the acceptable range of 90–110%, confirming the accuracy of the method.

#### 2.6.5. Quality Control and Data Analysis

Experimental samples were run in triplicate for the entire experiment. The effect of the background matrix was controlled by running replicate blanks in each case. All experimental samples were acidified using a drop of 2% HNO_3_ (20 μL) for ICP analysis when there was a need to wait for an extended time over 24 hrs. Continuous calibration verifications (CCVs) were performed periodically, after every 10-sample analysis, and a maximum 5% deviation of the CCV was accepted. The recovery of internal standards was in the range of 90% to 110%. Data analyses and graph plotting were conducted using Excel software (Version 2503 Build 16.0.18623.20178). The results were processed and indicated with a significant level of 5% (*p* < 0.5).

## 3. Results and Discussion

### 3.1. Physicochemical Properties of Column Fill C&DW Fines

**Figure 7:**
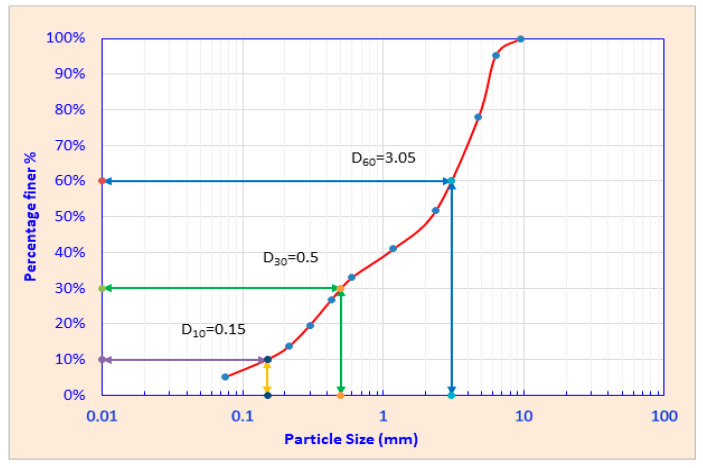
Grain size distribution of C&DW fine residue stockpiles from MRFs.

Sieve analysis of the C&DW fine residues revealed that about 80% of the samples were less than 4.75 mm in size (Figure 7). According to Australian standards for soil classification for engineering purposes (AS 1289.3.6.1-2009) [45], the coefficient of curvature for a well-graded soil, $C_{c}=\left[\frac{{\left(D_{30}\right)}^{2}}{D_{60}\times D_{10}}\right]\;$ should fall between 1 and 3, while its uniformity coefficient, $C_{u}=\frac{D_{60}}{D_{10}}$, should be beyond 4 for gravel or 6 for sand [46,47]. The C&DW fine residues were categorized as poorly graded soil with Cc of 0.4575 and Cu of 20.33.

This finding highlights the finer composition of C&DW fine residues in Australia, which can be attributed to the use of automated C&DW processing facilities that crush and mix various waste components, resulting in more uniform finer fractions. These results are consistent with the size characteristics of C&DW fine residues observed in Japan, Italy, and Portugal [48,49,50]. As specified in American Society for Testing and Materials and Australian Standards [45,51,52], the recommended particle size for materials undergoing leaching tests is less than 4.75 mm. Since over 80% of the samples fall within this size range, the leaching test results can be considered representative of typical C&DW samples.

The pH of the leachate ranged from 5.05 (observed in Weeks 19 and 21 for the 100% organic waste stream) to 7.47 (recorded in the first week for the 100% C&DW fine fractions stream). This pH range is relatively lower than those reported in previous studies on various types of C&DW. For instance, Somasundaram et al. [53] observed pH levels between 5.3 for the wooden fraction and 11.4 for the concrete fraction of C&DW, while mixed C&DW typically ranged from 9.6 to 9.9. In another study, Saca et al. [54] reported that the pH of concrete samples ranged from 8.45 to 11.82. Additionally, a column leaching experiment by Min et al. [55] using mixed C&DW reported pH values from 5.6 to 10.8. The lower pH levels observed may result from differences in C&DW composition across countries. For instance, Australia, as one of the world’s largest consumers of gypsum board [56,57,58,59], has a significant portion of residential buildings made from wood and gypsum board. In contrast, South Korea, which consumes minimal gypsum board in construction, favors concrete and other durable materials for its residential properties, as reflected in the studies above [60]. Such variations in materials likely contribute to differences in the pH profiles of C&DW. This suggests that the C&DW in the present study contains a significant amount of wood and gypsum board fragments within the mixed waste. With a pH of 4.5 for the leaching fluid fed into the columns and a leachate pH of 5.05 for the 100% organic waste column, the slightly higher pH of 7.47 for the 100% C&DW stream indicates that C&DW possesses acid-neutralizing capacity. This characteristic suggests its potential application in treating low-pH industrial discharges and naturally acidic environments [61,62,63,64].

**Figure 8:**
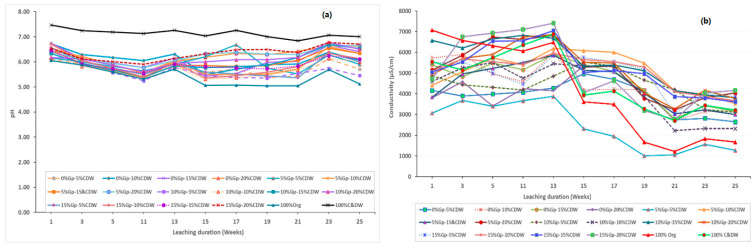
pH (**a**) and conductivity (**b**) of leachate as a function of leaching duration (NB: 0-5-95 and 5-5-95, etc., signify the corresponding concentrations of 0% Gyp, 5% C&DW, and 95% Org Waste, and 5% Gyp, 5% C&DW, and 95% Org Waste, respectively).

**Figure 9:**
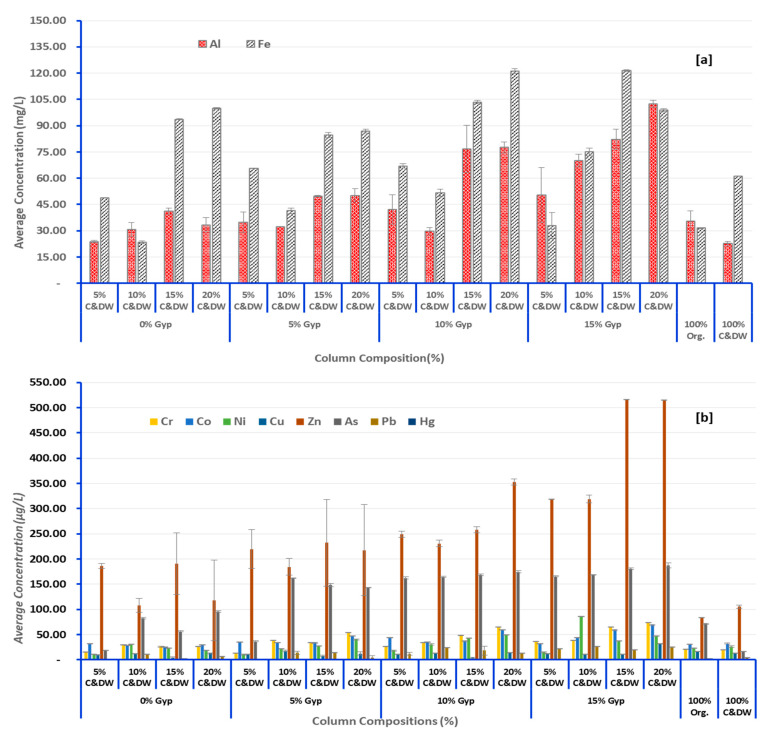
Average concentration of heavy metals in the leachate during the maximum leaching week [Week 13]. (**a**)—mg/L and (**b**)—µg/L.

Overall, the different column compositions have shown nearly similar pH patterns for the first 13 weeks, with slight variations occurring afterwards (Figure 8a). This change over time as conclusively argued by different researchers [65,66,67,68] could be attributed to the progressive depletion over time in the chemical composition of the column contents, essentially a characteristic of the continuous leaching process and variation in column compositions. With respect to the pH and conductivity curves over time, similar leaching patterns have been reported for different waste materials at various pH ranges with higher leaching rates at both extreme pH values and lowest leaching rates at or near neutral pH [69,70,71]. Conductivity initially ranged from 3000 to 7000 µS/cm at the beginning of the experiment and gradually decreased to 1000–4000 µS/cm towards the end, aligning with the findings of Hyks et al. [65] (pp. 522–529), Hyks et al. [66] (pp. 80–91), Dijkstra [67], and Nakao et al. [68]. Moreover, a notable change in conductivity was observed from about the 13th to 15th week onwards. This suggests a corresponding depletion of dissolved minerals from this time onwards as observed in the variation of pH values from the 13th week onwards. This notable change in conductivity aligns with the cumulative concentration of heavy metals observed, as depicted in Figure 9. The figure demonstrates a steady increase in heavy metal concentrations up to the 13th to 15th week, followed by a relatively stable trend thereafter. Columns with relatively higher proportions of gypsum and C&DW content (15% Gyp-15% C&DW, 15% Gyp-20% C&DW, and 100% C&DW) recorded relatively higher conductivity levels, indicating a sustained release of dissolved minerals over the long term.

### 3.2. Heavy Metals of Concern Leached from C&DW

**Figure 10:**
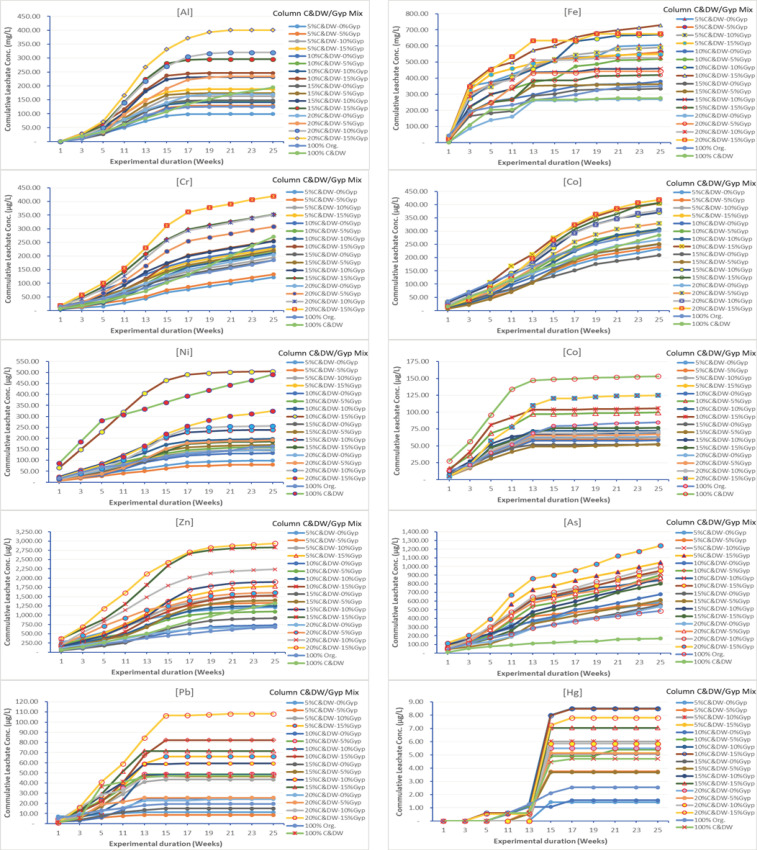
The cumulative concentration of heavy metals in leachate across different levels of C&DW and gypsum content.

**Figure 11:**
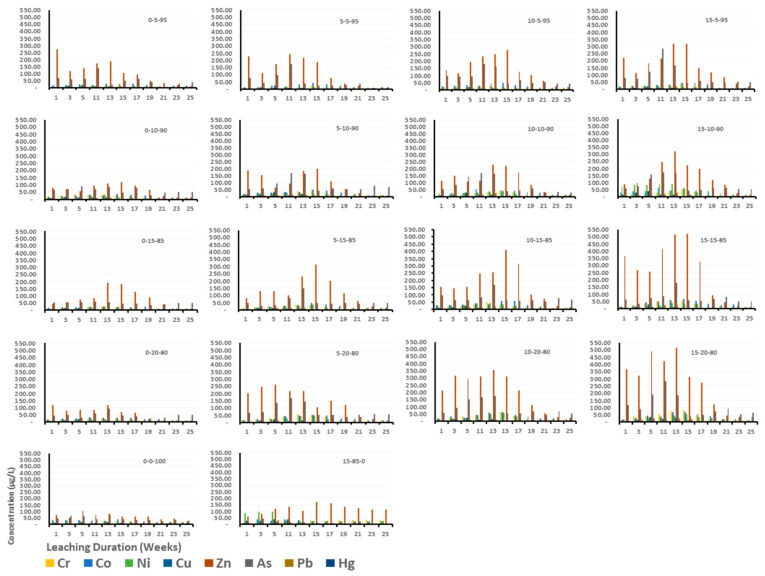
Temporal dynamics of heavy metal concentrations (µg/L) across different C&DW proportions (0-5-95 => Gyp-C&DW-Org).

Table 2 and Figure 9, Figure 10 and Figure 11 provide data on heavy metals in the leachates across various columns fill proportions of C&DW with different levels of gypsum content. For most elements, the lowest concentration observed was at 5% C&DW without gypsum, while the highest concentration was recorded at 20% C&DW with 15% gypsum. Nevertheless, none of the column mixes exceeded the maximum allowable values for leachable concentration or specific contaminant concentration (SCC) for general and restricted solid wastes (Table 2) [72]. Although metals like Aluminum (Al) and Iron (Fe) are not included in the Toxicity Characteristics Leaching Procedure (TCLP) and SCC guideline values for waste classification due to their natural abundance in soils and perceived lower environmental risk, they, however, exceeded the maximum permissible levels set by the Australian and New Zealand guidelines for fresh and marine water quality [73]. The relatively elevated concentrations of Al and Fe recorded in this study indicate that the source of C&DW fines is composed of brick fragments. This conclusion is corroborated by X-ray fluorescence analysis conducted by Gao et al. [74], which identified the typical chemical compositions of brick-based C&DW as SiO_2_ (30.2–60.5%), Al_2_O_3_ (4.6–18.9%), and Fe_2_O_3_ (1.5–7.6%), aligning with our findings. Diotti et al. [75] on his part reported that fine residues and recycled aggregates from C&DW are rich in Si, Al, and Fe with concentrations of 421,000 mg(Si)/kg, 12,021 mg(Al)/kg, 211.30 mg(Fe)/kg, respectively. Furthermore, the material profiles of C&DW delivered to recovery facilities in Australia, predominantly originating from demolition activities, are primarily comprised of mixed C&DW fractions, with bricks and concrete making up the majority [76]. Furthermore, elevated levels of Al and Fe in the leachate may also result from cross-contamination caused by mechanized demolition, inadequate segregation practices, and crushing during waste processing activities [22].

On the other hand, Pb, Hg, As, Cr, Ni, Cu, Zn, and Co were heavy metals detected in this study. Nevertheless, none of these heavy metals exceeded the maximum values for leachable concentration and SCC for general and restricted solid wastes [72]. Saca et al. [54] reported comparable patterns of heavy metals in C&DW leachate, with concentrations falling below the limits established by European regulations for inert waste acceptance at landfills. The low concentrations of heavy metals observed in the leachates could be attributed to several factors. Firstly, it may reflect effective waste segregation practices within the waste management system or an inherently low level of heavy metal content in the waste streams that constitute the C&DW fines during the study period. Secondly, sulfate-reducing bacteria (SRB) formation due to the prevailing anaerobic conditions in the leaching column might play a significant role. These anaerobic conditions facilitate the formation of metal sulfides, which bind the metals, reducing their solubility and subsequent detection in the leachate [55,77,78]. Moreover, the organic matter within the columns formed heavy metal–organic matter complexes, which affects the solubility of heavy metals and, in effect, reduces the concentration of heavy metals within the leachate [79]. Despite this, the findings show a consistent increase in heavy metal concentrations with higher gypsum and C&DW contents, underscoring the potential environmental risks posed by C&DWs.

### 3.3. Effect of C&DW Content on the Concentration of Heavy Metals in the Leachate

**Figure 12:**
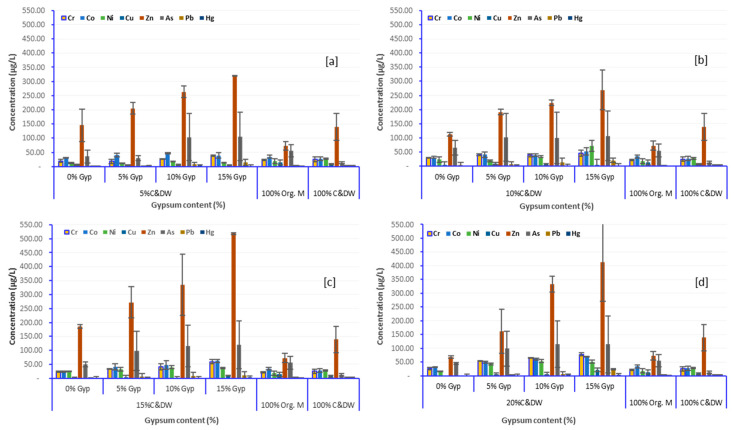
Effect of C&DW ((**a**)—5%, (**b)**—10%, (**c**)—15%, and (**d**)—20%) and gypsum content (0, 5, 10, and 15%) on leaching behavior of heavy metals during the peak leaching period (Week 13).

The results presented in Table 2 and Figure 9, Figure 10, Figure 11 and Figure 12 highlight the concentrations of heavy metals in the leachate across different column fill combinations. Figure 10 and Figure 11 illustrate the overall trends, while Table 2 and Figure 9 focus on the 13th week of leaching, identified as the peak leaching period. This week provides a representative snapshot of the maximum environmental impact caused by the leaching of C&DW. The data reveal that heavy metal concentrations in the leachate increased with higher proportions of C&DW and its associated gypsum content.

As shown in Figure 10 and Figure 11, and further supported by the pH and conductivity patterns observed in Figure 8, the C&DW content had a significant impact on the concentration of heavy metals in the leachate throughout the experimental period. Low-C&DW mixtures, such as 0-0-100, exhibited significantly lower metal concentrations, with only minor fluctuations observed during the early weeks. Conversely, medium and high-C&DW mixtures, such as 15-10-85 and 15-85-0, saw sharp early spikes followed by sustained higher levels of contamination. Figure 10 clearly illustrates that higher C&DW content is directly associated with an increased concentration of heavy metals, including Pb, Hg, As, Cr, Ni, Cu, Zn, and Co, as well as other metals such as Al and Fe. Mixtures with a higher proportion of C&DW consistently demonstrated elevated cumulative concentrations compared to those with lower C&DW content alone or gypsum mixtures. Metals such as Fe and Zn were particularly affected, exhibiting notably steep increases in cumulative concentrations as C&DW content rose, highlighting their heightened sensitivity to C&DW content.

In addition to an overall rise in leachate concentrations, analysis of the temporal behavior of heavy metals leaching highlights the direct relationship between the proportion of C&DW in the columns and the intensity of metal leaching. This leaching pattern generally follows a two-phase trend. In the initial weeks, a rapid increase in heavy metal concentrations is observed particularly within the first 13 to 15 weeks of the experiment. This is subsequently followed by a gradual decline, indicating a transition to continuous but reduced leaching. This initial surge is attributed to the mobilization of surface-bound and highly soluble contaminants present in C&DW. This finding also aligns with those of Diotti et al. [80], who noted that the leaching of heavy metals from C&DW is primarily influenced by the solubility of surface-deposited metals and their interactions with water during the early stages of the leaching process. Similarly, Zhang et al. [81] reported that the rapid release of contaminants in the initial weeks corresponds to the dissolution of easily accessible fractions, such as metal oxides, paint residues, and corrosion products. Further supporting this observation, Van Praagh and Modin [82] and Townsend et al. [83] highlighted the role of soluble contaminants in driving these initial peaks, emphasizing that the material composition of C&DW—such as painted surfaces, treated wood, and galvanized components—contribute heavy metals such as Pb, Zn, and Hg among others. Furthermore, the magnitude of these early spikes is directly proportional to the percentage of C&DW in the mixture, with higher proportions of C&DW leading to much larger initial concentrations. These initial release patterns underscore the critical need to understand leaching dynamics during the early stages to mitigate the environmental risks associated with C&DW leachates, a concern also emphasized by Chen et al. [78], Mondal et al. [84], Eckbo et al. [85], and Rubinos and Spagnoli [86].

Following this peak, a noticeable decline in the concentration of these chemical species was recorded, indicating the exhaustion of soluble materials or the stabilization of the waste matrix. Over time, anaerobic conditions developed within the leaching column, fostering the proliferation of sulfate-reducing bacteria (SRB) [78,87,88,89]. These bacteria promote the formation of metal sulfide precipitates, which reduce heavy metal solubilization and lower their concentrations in the leachate [55,77,81]. This transition highlights the interplay between biogeochemical processes and waste composition in influencing long-term leaching behavior.

Aluminum and iron increased their cumulative concentration with higher C&DW mixtures, resulting in substantially greater total concentrations. This behavior is directly related to their high presence in construction materials and their reactivity under various conditions. Studies, such as those by Diotti et al. [75], emphasize the influence of pH-dependent processes and waste composition on metal release dynamics. For example, aluminum, being amphoteric, reacts in both acidic and basic environments, contributing to its steady leaching behavior distinctly delineated in higher proportions such as 15% and 20% C&DW among others [90,91,92].

In contrast, toxic metals like Cr, As, Pb, and Hg also exhibit cumulative increases, though their concentrations tend to stabilize more consistently as the pH stabilizes to neutrality. Moreover, the intrinsic chemical stability of these toxic metals in various environmental matrices and their interactions with stabilizing agents in waste could contribute to their tendency to for precipitates. Wang et al. [93] highlighted that arsenic forms calcium arsenate hydrates (Ca_3_(AsO_4_)_2_·xH_2_O), which are less soluble under typical landfill conditions. Similarly, lead can bind to sulfides or hydroxides [94], while mercury stabilizes through binding with organic matter or sulfide minerals [95,96]. Despite their chemical stability, these metals remain toxic due to their propensity for bioaccumulation and biomagnification. They concentrate within organisms and amplify through the food chain, resulting in toxic effects even at low concentrations [97,98]. Additionally, environmental changes can also alter the chemical forms of these metals, enhancing their mobility and bioavailability, which further heightens their toxic potential [99]. These metals are also non-biodegradable, contributing to long-term ecological and health risks, as highlighted in studies by Ali and Khan [100] and Zaynab et al. [101], underscoring the critical need for effective mitigation strategies to reduce their detrimental impacts.

As illustrated in Figure 10, the cumulative concentration of mercury (Hg) exhibited a sharp increase between weeks 13 and 15, a trend that deviated from the behavior of other elements. This irregularity may be attributed to several factors. Firstly, the extremely low concentrations of mercury observed in multiple column setups over time may have resulted in spurious or inconsistent associations. On the other hand, but more importantly, in environments such as landfills—organic matter and sulfide rich environments—mercury is predominantly immobilized through the formation of mercuric sulfide (HgS), a highly insoluble compound that significantly limits mercury’s detectability in leachates [102]. However, as the microbial degradation of protein-rich organic matter progresses, it releases compounds such as polysulfides and cysteine. These sulfur-containing ligands can interact with HgS, converting it into more soluble species, such as Hg^2+^ and S^2−^. In addition, the displacement of Hg^2+^ by other metals with a strong affinity for sulfur—such as iron (Fe) and copper (Cu)—can promote the dissolution of HgS. Microorganisms themselves can also mediate HgS dissolution over time [103,104]. As microbial activity intensifies and organic matter continues to degrade, these combined biogeochemical processes can lead to a delayed increase in mercury concentrations observed at later stages of the leaching experiment.

Unlike toxic metals that tend to remain relatively immobile, elements like iron and zinc show varied stabilization behaviors depending on their chemical interactions with redox-sensitive environments. Specifically, iron oxides are sensitive to changes in oxygen levels. Under reducing (low oxygen) conditions, these oxides can dissolve, releasing previously adsorbed or bound contaminants. When oxygen becomes available again (oxidizing conditions), the dissolved iron can re-precipitate as solid oxides. This cycle of dissolution and re-precipitation leads to inconsistent or non-linear patterns in how iron and its associated contaminants are stabilized or mobilized over time. In contrast, zinc behaves differently. It becomes highly soluble in acidic environments, such as those that may develop during waste degradation, and readily forms complexes with dissolved organic matter or other ligands in the leachate [105]. These soluble zinc complexes are more mobile and thus more easily leached out from the material. Overall, the leaching trends presented in Figure 10 and Figure 11 clearly indicate that columns with high contents of C&DW—particularly those with more than 10% C&DW and specifically those containing 15% gypsum (15-10-85, 15-15-85, and 15-20-80)—exhibited significantly elevated leachate concentrations throughout the experimental period. This notable difference highlights the critical role of waste composition in influencing long-term metal mobility and its potential environmental consequences.

Metals like zinc and lead show stark differences between mixtures with low and high C&DW content, with the latter contributing significantly higher concentrations throughout the experimental period. This relationship highlights the pronounced impact of C&DW on heavy metal mobilization, as detailed by Molla et al. [22], that documented a review of similar concentration patterns in systematic leaching studies. As argued by Diotti et al. [80], Robey et al. [106], and Townsend et al. [83], early mobility of heavy metals is not only governed by C&DW content but also by pH, particle size, and the presence of organic matter. Robey et al. [106] specifically underscored that C&DWs facilitate the early and significant mobilization of metals like lead and mercury, potentially posing environmental and health risks when improperly managed.

Generally, while most metals initially exhibit elevated leaching concentrations that stabilize over time, some show significantly higher leaching levels and respond strongly to increases in C&DW content. Metals such as zinc demonstrate more consistent weekly leaching patterns but still display higher cumulative concentrations in environments with increased C&DW proportions, emphasizing their strong dependence on material composition. The overall pattern observed over 25 weeks suggests that C&DW content not only influences the magnitude of metal release but also its temporal characteristics. The sharp release of metals in the initial weeks indicates a potential for immediate environmental impact, while the sustained cumulative increases highlight longer-term risks.

In particular, higher C&DW content significantly enhances the release of heavy metals into the leachate, with metals such as lead and zinc being especially affected. These metals exhibit both high initial spikes and substantial cumulative concentrations, underscoring the environmental risks posed by the improper disposal or management of C&DW. Effective waste management strategies are, therefore, essential to mitigate these risks and reduce the potential for environmental harm.

### 3.4. Effect of Gypsum on Leaching Characteristics of Heavy Metals

Variations in gypsum content significantly impacted leachate chemistry in columns containing C&DW and organic material. This phenomenon arises from several interrelated factors. Firstly, gypsum ($Ca{SO}_{4}\cdot 2H_{2}O$) interacts with water to release sulfate ions. Moreover, the initial low pH feed water (rain water) enhances this dissolution to some degree according to the following reaction [107]:

(1) $$Ca{SO}_{4}\cdot 2H_{2}O⇌{Ca}^{2+}+{{SO}_{4}}^{2-}+{2H}_{2}O$$

Moreover, the organic waste component within the landfill (e.g., food scraps, paper, and yard waste) undergoes anaerobic degradation, producing organic acids, CO_2_, and hydrogen sulfide (H_2_S), which themselves can contribute to acidic environment.

(2) $${SO}_{4}^{2-}+{2CH}_{2}O+{2H}^{+}\overset{SRB}{\to }H_{2}S+{2CO}_{2}+{2H}_{2}O$$

where CH_2_O represents organic matter. On the other hand, the breakdown of proteins and other sulfur-containing organics also releases sulfide (S^2−^) [81,108], all of which contribute to weak acid solutions. Furthermore, the carbonic acid from the organic matter dissociates to release hydrogen and carbonate ions.

(3) $${CO}_{2}+H_{2}O⇋H_{2}{CO}_{3}$$

(4) $$H_{2}{CO}_{3}⇋{{HCO}_{3}}^{-}+H^{+}$$

(5) $${{HCO}_{3}}^{-}⇋{{CO}_{3}}^{2-}+H^{+}$$

The carbonate ion can react with calcium ion in gypsum to form insoluble calcium carbonate ${{CO}_{3}}^{2-}+{Ca}^{2+}⇋Ca{CO}_{3}$. As the anoxic decomposition of organic matter proceeds, more carbon dioxide becomes available, resulting in formation of more carbonate ion. This process enhances the formation of calcium carbonate subsequently resulting in more dissolution of gypsum. This process not only dissolves gypsum but also results in an increased $H^{+}$ and ${{SO}_{4}}^{2-}$ concentration in solution, which might contribute to bisulfate (HSO_4−_) formation.

(6) $${{SO}_{4}}^{2-}+H^{+}⇋{{HSO}_{4}}^{-}$$

Bisulfate (HSO_4_^−^) is a weak acid, contributing to acidity of the system. In effect, the overall process played a key role in creating acidic conditions in the waste environment [108,109]. This low-pH environment enhances the solubility of heavy metals, thereby increasing their mobility [110]. Secondly, the sulfate ions produced by gypsum coupled with dissolved organic matter from the degrading organic components can form soluble metal-sulfate complexes with certain heavy metals, such as lead (Pb) and zinc (Zn), which are more soluble in water than the metals in their native forms [89,94,111]. Additionally, gypsum can participate in chemical reactions with other minerals or compounds in the waste, leading to the release of previously immobilized heavy metals [112,113]. Finally, gypsum also influences redox conditions within the waste matrix, facilitating the oxidation or reduction of heavy metals and further promoting their release into leachate [114,115].

Higher gypsum content increased the concentration of sulfate and other soluble ions in the leachate, raising its ionic strength and conductivity as shown in Figure 8b [78]. On the other hand, when gypsum content remains constant, an increased proportion of C&DW leads to a greater release of soluble materials, resulting in higher concentrations of various chemical species. This could be attributed to the enhanced surface area for chemical reactions and the increased availability of inorganic and organic chemicals for leaching, contributing to the overall complexity and variability of the leachate composition [116]. Moreover, as can be seen from Figure 10 and Figure 11, relatively higher concentrations and cumulative concentrations of heavy metals were recorded with columns having higher gypsum content.

Figure 12 illustrates the influence of gypsum content (0, 5, 10, and 15%) on heavy metal leaching across varying C&DW proportions (a, b, c, and d). Furthermore, it shows a clear trend of increasing metal concentrations as gypsum content rises from 0% to 15%. Zn and As exhibited notable increases with rising gypsum levels, especially in mixtures with higher C&DW content, consistently showing the highest concentrations compared to other metals. Nurhanim et al. [117] highlighted that Zn and As consistently exceeded other metals in leachates, underscoring their sensitivity to gypsum-enhanced leaching processes. Tayibi et al. [118] corroborated these findings, identifying Zn and As as the most prominent metals leached from gypsum-containing waste mixtures. On the other hand, other metals such as Hg, Pb, Co, Ni, and Cu exhibit relatively lower concentrations in the leachates. However, slight increases in their levels were also evident with rising gypsum content. These results highlight the cumulative effect of gypsum and C&DW proportions in amplifying heavy metal leaching, with the most pronounced effects observed at 15% gypsum content.

Quantitative data from Figure 12 provide further evidence of this trend. In mixtures containing 5% and 10% C&DW, a gypsum content of 15% resulted in maximum Zn concentrations of 319.3 µg/L and 319 µg/L, while As concentrations reached 165.8 µg/L and 168.7 µg/L, respectively (Figure 12a,b). At higher C&DW proportions of 15% and 20%, the same gypsum content elevated Zn concentrations to 516.0 µg/L and 514.8 µg/L, while As concentrations rose to 181.0 µg/L and 187.2 µg/L, respectively (Figure 12c,d). These observations, also reflected in Figure 9, highlight the significant role of gypsum in enhancing heavy metal leaching. In contrast, control samples composed entirely of organic material or 100% C&DW exhibited substantially lower metal concentrations, underscoring the pronounced effect of gypsum on heavy metal release.

These findings emphasize gypsum’s role in enhancing heavy metal mobilization, particularly in settings with higher C&DW proportions, resulting in elevated concentrations of all heavy metals compared to mixtures with lower levels of C&DW and gypsum. These findings are corroborated by studies such as those by Chen et al. [78] and Diotti et al. [75], which highlight the role of gypsum in enhancing heavy metal leaching from C&DW, particularly under conditions where gypsum and C&DW proportions are high. Similarly, research by Lee et al. [119] and Shruthi et al. [111] supports these observations, showing that gypsum increases metal solubility and facilitates the mobilization of contaminants like Zn and As, providing a detailed understanding of how gypsum alters chemical equilibria and pH conditions, thereby intensifying metal release. This aligns closely with the results reported in this study.

## 4. Conclusions and Recommendation

This study investigated the leaching behavior of C&DW fine residues, focusing on the release of heavy metals under varying proportions of gypsum and organic content. The results highlight significant environmental challenges associated with C&DW disposal, particularly in landfill settings. It also provides a comprehensive analysis of the physicochemical properties of C&DW fines, particularly in relation to their particle size, leachate chemistry, and heavy metal mobilization, emphasizing the environmental implications of their management. Over 80% of C&DW fines were smaller than 4.75 mm, technically classified as poorly graded soil. Their physicochemical characteristics, including a pH range of 5.05 to 7.47 and a conductivity decline from 3000–7000 µS/cm to 1000–4000 µS/cm, indicate mineral depletion during leaching. Temporal changes in pH and conductivity highlight ongoing chemical processes, especially after the 13th week of leaching, indicating its long-term impact.

Leaching analysis revealed a two-phase behavior: an initial rapid release of heavy metals during the first 13–15 weeks, followed by stabilization as soluble surface-bound contaminants were depleted and anaerobic conditions promoted metal stabilization. Al and Fe, despite not being considered as elements of concern for waste classification tests, exceeded the maximum permissible levels set by the Australian and New Zealand Guidelines for Fresh and Marine Water Quality, highlighting its potential short-term impacts. Most heavy metals such as Pb, Hg, As, Cr, Ni, Cu, Zn, and Co, on the other hand, remained within acceptable regulatory thresholds for general or restricted solid waste classification. Nevertheless, the distinct leaching patterns observed (higher combinations of C&DW and gypsum content consistently leading to increased heavy metal concentrations in leachate, with most toxic metals exhibiting early spikes and long-term cumulative increases) across different column mix combinations highlight the role C&DW can play in contributing to long-term environmental impacts. Importantly, the potential for toxic metals to bioaccumulate and persist in the environment should not be underestimated. Our findings—supported by results from previous studies—underscore that landfilling C&DW fine residues poses a non-negligible environmental risk over time. Even trace levels of such hazardous metals can accumulate in soil and groundwater, gradually affecting ecological and human health—particularly in regions with less regulated waste management or sensitive groundwater systems.

Gypsum content was a key factor in enhancing heavy metal mobilization. Its interaction with water reduced pH, increasing ionic strength, and promoted the formation of soluble metal-sulfate complexes, particularly for metals such as Zn and As. Columns with higher gypsum and C&DW proportions—specifically mixtures containing 15% and 20% C&DW combined with 10% and 15% gypsum—exhibited significantly elevated heavy metal concentrations in the leachate. These findings underscore the synergistic impact of gypsum in C&DW and Organic matter on leaching dynamics and highlight the potential environmental risks associated with disposing of waste mixtures with such compositions. The study also highlights the environmental hazards associated with improper disposal of C&DW and gypsum, particularly during early leaching stages when soluble contaminants are most active, and over the long term due to the bioaccumulative nature of many toxic heavy metals.

To mitigate these impacts, it is imperative to improve waste segregation at demolition sites, adopt advanced material recovery facilities, and encourage the reuse of non-hazardous C&DW fractions in construction projects. Sustainable alternatives to gypsum should be explored alongside the implementation of advanced landfill containment systems and rigorous monitoring programs to prevent contamination. Policymakers must establish stricter guidelines for C&DW disposal and mandate regular environmental assessments to evaluate and mitigate ecological risks. Public awareness campaigns and research into low-impact building materials are also essential to support sustainable waste management practices and foster a circular economy. These strategies collectively provide a pathway to minimize the environmental risks of C&DW disposal and enhance resource recovery.

## Figures and Tables

**Table 1:** Column mix proportions and arrangement.

Column ID No.	C&DW Content (%)	Gypsum Content (Gyp %)	Max. Organic Content (Org %)	Remarks
1–4	5	0, 5, 10 and 15	95%	Test columns
5–8	10		90%	Test columns
9–12	15		85%	Test columns
13–16	20		80%	Test columns
17	0	0	100	Control-Org
18	85	15	0	Control-C&DW

**Table 2:** Average concentration of heavy metals in the leachate samples (measured in µg/L) during the maximum leaching week [Week 13 (other metals like Al and Fe measured in mg/L)].

Column Fill (%)		Metal Concentration, ±STDVE									
C&DW	Gypsum	Al * [20]	Fe * [20]	Cr * [100]	Co * [1000]	Ni * [2000]	Cu * [200]	Zn * [20,0]	As ** [5000]	Pb ** [5000]	Hg ** [200]
5% C&DW	0	23.9 ± 0.77	48.8 ± 0.04	15.2 ± 0.36	31.8 ± 0.42	11.2 ± 0.42	10.0 ± 0.45	186.4 ± 4.63	18.8 ± 0.29	0.9 ± 0.80	<RL ± 0.03
	5	34.9 ± 5.87	65.7 ± 0.00	13.2 ± 0.32	35.3 ± 0.35	10.4 ± 0.21	11.0 ± 0.41	219.5 ± 14.24	36.6 ± 1.66	1.1 ± 0.22	0.7 ± 0.00
	10	42.2 ± 8.28	67.0 ± 1.37	27.4 ± 0.86	44.8 ± 0.14	18.2 ± 0.09	11.8 ± 0.74	249.0 ± 61.00	162.2 ± 0.83	12.0 ± 0.04	0.6 ± 0.01
	15	50.4 ±15.64	32.9 ±7.52	36.7 ± 0.17	31.8 ± 0.48	15.1 ± 0.03	12.4 ± 0.85	319.3 ± 80.37	165.8 ± 1.80	22.7 ± 0.06	<RL ± 0.00
10% C&DW	0	30.7 ± 4.03	23.5 ± 0.90	30.4 ± 0.23	28.9 ± 0.32	31.0 ± 0.83	12.1 ± 0.32	108.0 ± 38.54	83.0 ± 1.22	11.0 ± 0.13	0.5 ± 0.01
	5	32.3 ± 0.10	41.4 ± 1.59	38.3 ± 0.40	34.3 ± 0.36	21.8 ± 0.86	17.7 ± 1.34	184.6 ± 16.81	162.4 ± 0.64	13.7 ± 3.12	0.6 ± 0.00
	10	29.7 ± 2.17	51.6 ± 2.06	34.2 ± 0.43	36.0 ± 1.04	31.0 ± 0.22	12.9 ± 0.97	230.6 ± 86.23	164.5 ± 3.20	24.6 ± 0.94	0.7 ± 0.01
	15	70.1 ± 3.75	75.1 ± 2.24	39.1 ± 0.01	44.0 ± 0.46	85.7 ± 1.07	11.5 ± 3.47	319.0 ± 90.43	168.7 ± 0.76	26.8 ± 4.36	<RL ± 0.03
15% C&DW	0	41.1 ± 1.93	93.6 ± 0.28	25.9 ± 0.01	25.1 ± 0.29	24.0 ± 0.73	4.8 ± 0.05	190.6 ± 6.27	56.6 ± 2.14	2.2 ± 3.09	<RL ± 0.01
	5	49.8 ± 0.55	84.6 ± 1.52	33.8 ± 0.27	34.0 ± 0.28	27.3 ± 1.40	8.0 ± 0.46	232.2 ± 6.53	148.4 ± 1.32	14.1 ± 0.62	0.5 ± 0.00
	10	76. 8 ±13.39	103.4 ± 0.91	48.4 ± 0.54	37.2 ± 1.15	43.2 ± 0.26	4.0 ± 0.83	257.9 ± 5.76	168.6 ± 1.21	18.2 ± 8.63	<RL ± 0.05
	15	82.2 ± 5.63	121.4 ± 0.37	64.7 ± 0.05	59.0 ± 0.39	37.8 ± 0.09	11. 6 ± 0.10	516.0 ± 6.53	181.0 ± 1.74	20.1 ± 0.56	<RL ± 0.00
20% C&DW	0	33.4 ± 4.19	100.0 ± 0.46	26.9 ± 0.21	30.0 ± 0.52	19.5 ± 1.07	12.7 ± 0.15	117.9 ± 0.43	96.2 ± 1.00	7.3 ± 0.02	<RL ± 0.02
	5	50.2 ± 3.73	86.8 ± 1.14	54.8 ± 0.12	46.9 ± 0.43	40.4 ± 0.84	12.2 ± 0.13	217.6 ± 7.66	143.2 ± 0.41	4.1 ± 0.13	<RL ± 0.01
	10	77.6 ± 3.14	121.1 ± 1.38	65.9 ± 0.31	59.3 ± 0.93	49.9 ± 0.19	14.2 ± 0.10	352.8 ± 0.61	174.7 ± 1.60	12.8 ± 0.05	<RL ± 0.02
	15	102.4 ±2.03	98.9 ± 0.73	73.7 ± 0.37	69.1 ± 1.21	47.0 ± 0.38	31.9 ± 0.08	514.8 ± 0.31	187.2 ± 4.84	25.5 ± 0.33	<RL ± 0.00
100% Org. W		26.4 ± 35.37	6.0 ± 31.57	0.0 ± 20.81	0.4 ± 20.81	0.2 ± 30.94	0.2 ± 24.06	0.0 ± 16.73	0.0 ± 83.79	0.6 ± 71.26	1.2 ± 2.95
100% C&DW		23.1 ± 22.73	1.3 ± 61.27	0.0 ± 19.61	0.1 ± 19.61	0.2 ± 32.39	0.7 ± 26.00	1.8 ± 13.15	0.3 ± 105.37	3.0 ± 16.21	0.4 ± 4.22

* Australian and New Zealand guidelines for fresh and marine water quality [73]; ** maximum values of leachable concentration and specific contaminant concentration for general waste [72].

## Data Availability

The data that support the findings of this study are openly available in the University of Newcastle’s digital data repository at http://hdl.handle.net/1959.13/1517578, reference number uon:57131, available online from 10 February 2025. The dataset includes all raw and processed data from the column leaching experiments, including leachate chemistry, heavy metal concentrations, and physicochemical properties of the C&DW fine residues. Additional supporting information, such as experimental protocols and analytical methods, can be provided to ensure reproducibility and transparency. For further inquiries or access to supplementary materials, please contact the principal investigator.

## Abbreviations

The following abbreviations are used in this manuscript:

ABBA	Australian Biomass for Bioenergy Assessment
ARENA	Australian Renewable Energy Agency
C&DW	Construction and Demolition Waste
EPA	Environmental Protection Authority
GCER	Global Centre for Environmental Remediation
HPIC	High-Pressure Ion Chromatography
ICP-MS	Inductively Coupled Plasma Mass Spectroscopy
MRF	Material Recovery Facility
MSW	Municipal Solid Waste
NIER	Newcastle Institute for Energy and Resources
NSW	New South Wales
ORS	Octapole Reaction System
ppt	Part per trillion
SCC	Specific Contaminant Concentration
SRB	Sulfate-Reducing Bacteria
TCLP	Toxicity Characteristics Leaching Procedure
UPVC	Unplasticized Polyvinyl Chloride

## References

[1] Ritchie H., Rodés-Guirao L., Mathieu E., Gerber M., Ortiz-Ospina E., Hasell J., Roser M. (2024). Population Growth. https://ourworldindata.org/population-growth?insight=the-unexpects-the-global-population-to-peak-by-the-end-of-the-century#article-citation.

[2] Worldometers.info. World Population Clock: 8.12 Billion People (2024)—Worldometer. https://www.worldometers.info/world-population/.

[3] Mo K.H., Alengaram U.J., Jumaat M.Z. (2016). Structural performance of reinforced geopolymer concrete members: A review. Constr. Build. Mater..

[4] Eriksson P., Milić V., Brostrom T. (2019). Balancing preservation and energy efficiency in building stocks. Int. J. Build. Pathol. Adapt..

[5] Gharehbaghi K., Rahmani F., Paterno D. (2020). Sustainable concrete in transportation infrastructure: Australian case studies. IOP Conf. Ser. Mater. Sci. Eng..

[6] Duan Z.H., Poon C.S. (2014). Properties of recycled aggregate concrete made with recycled aggregates with different amounts of old adhered mortars. Mater. Des..

[7] De Luca A., Chen L., Gharehbaghi K. (2020). Sustainable utilization of recycled aggregates: Robust construction and demolition waste reduction strategies. Int. J. Build. Pathol. Adapt..

[8] Pickin J., Wardle C., O’Farrell K., Nyunt P., Donovan S., Department of Agriculture, Water and the Environment (2020). National Waste Report 2020.

[9] Han D., Mohsen K., Abbas R. (2021). Building information modeling (BIM) for construction and demolition waste management in Australia: A research agenda. Sustainability.

[10] Duan H., Yu D.F., Zuo J., Yang B., Zhang Y.K., Niu Y.N. (2016). Characterization of brominated flame retardants in construction and demolition waste components: HBCD and PBDEs. Sci. Total Environ..

[11] Lingard H., Graham P., Smithers G. Waste management in the Australian construction industry: A human factors approach. Proceedings of the 13th Annual ARCOM Conference.

[12] Lingard H., Gilbert G., Graham P. (2001). Improving solid waste reduction and recycling performance using goal setting and feedback. Constr. Manag. Econ..

[13] Liu Y., Sun T., Yang L. (2017). Evaluating the performance and intellectual structure of construction and demolition waste research during 2000–2016. Environ. Sci. Pollut. Res..

[14] Carr A., Fetherston É., Meyer L., Makled T. (2019). Towards a Circular Plastics Economy: Policy Solutions for Closing the Loop on. Master’s Thesis.

[15] Tran T., Goto H., Matsuda T. (2021). The impact of China’s tightening environmental regulations on international waste trade and logistics. Sustainability.

[16] Shoostarain S., Maqsood T., Yang R., Khalfan M., Wong P. The Impact of New International Waste Policies on the Australian Construction and Demolition Waste Stream. Proceedings of the 44th AUBEA-Australasian Universities Building Education Association Conference.

[17] Browne K. Impacts of China’s ‘Green Sword’ Policy on Australia’s Waste Disposal. HG Lawyers. https://www.hopgoodganim.com.au/page/knowledge-centre/blog/impacts-of-chinas-green-sword-policy-on-australias-waste-disposal.

[18] Shooshtarian S., Maqsood T., Wong P.S.P., Malik K., Yang R.J. (2020). Landfill levy imposition on construction and demolition waste: Australian stakeholders’ perceptions. Sustainability.

[19] Sahajwalla V. (2018). Big challenges, micro solutions: Closing the loop in Australia’s waste crisis. AQ-Aust. Q..

[20] Shooshtarian S., Maqsood T., Wong P.S.P., Malik K., Yang R.J. (2020). Australian Construction and Demolition Waste Management System in Australia: Investigation of Challenges and Opportunities. Preprints.

[21] Duan H., Miller T.R., Liu G., Tam V.W.Y. (2019). Construction debris becomes growing concern of growing cities. Waste Manag..

[22] Molla A.S., Tang P., Sher W., Bekele D.N. (2021). Chemicals of concern in construction and demolition waste fine residues: A systematic literature review. J. Environ. Manag..

[23] Delay M., Lager T., Schulz H.D., Frimmel F.H. (2007). Comparison of leaching tests to determine and quantify the release of inorganic contaminants in demolition waste. Waste Manag..

[24] Jang Y.-C., Townsend T. (2001). Sulfate leaching from recovered construction and demolition debris fines. Adv. Environ. Res..

[25] Roussat N., Méhu J., Abdelghafour M., Brula P. (2008). Leaching behaviour of hazardous demolition waste. Nucl. Chem. Waste Manag..

[26] Jambeck J.R., Townsend T.G., Solo-Gabriele H.M. (2008). Landfill Disposal of CCA-Treated Wood with Construction and Demolition (C&D) Debris: Arsenic, Chromium, and Copper Concentrations in Leachate. Environ. Sci. Technol..

[27] Khan B.I., Jambeck J., Solo-Gabriele H.M., Townsend T.G., Cai Y. (2006). Release of Arsenic to the Environment from CCA-Treated Wood. 2. Leaching and Speciation during Disposal. Environ. Sci. Technol..

[28] Yu W., Saraya S., Hwidong K., Brajesh D., Timothy T. (2012). Mobilization of iron and arsenic from soil by construction and demolition debris landfill leachate. Waste Manag..

[29] Butera S., Christensen T.H., Astrup T.F. (2014). Composition and leaching of construction and demolition waste: Inorganic elements and organic compounds. J. Hazard. Mater..

[30] Powell J.T., Jain P., Smith J., Townsend T.G., Tolaymat T.M. (2015). Does disposing of construction and demolition debris in unlined landfills impact groundwater quality? Evidence from 91 landfill sites in Florida. Environ. Sci. Technol..

[31] New South Wales Government (2019). Protection of the Environment Operations (Waste) Regulation 2014, Section 12(7)(c): Recovered Fines Alternative Daily Cover Specifications. https://www.epa.nsw.gov.au/sites/default/files/NSWGG.2019.5.24.G53.pdf.

[32] NSW-EPA (2019). POEO Public Register, List of Licences. https://www.epa.nsw.gov.au/licensing-and-regulation/public-registers/about-prpoeo/list-of-licences.

[33] RENEW-NSW Regional Networks for Effective Waste Management. https://www.riverinawaste.nsw.gov.au/renew-nsw/.

[34] Cross S., Cross Connections Consulting (2016). ‘Bridging the Gap’ to divert plasterboard from landfill and reduce project costs. Waste Less, Recycle More Initiative.

[35] Australian Renewable Energy Agency Australian Renewable Energy Mapping Infrastructure. 13 August 2019. https://nationalmap.gov.au/renewables/.

[36] (2016). Standard Test Methods for Determination of the Composition of Unprocessed Municipal Waste.

[37] Lisa D., Anders L. (2008). Methods for household waste composition studies. Waste Manag..

[38] (2000). Methods for Sampling and Testing Aggregates.

[39] EPA-Victoria (2000). A Guide to the Sampling and Analysis of Waters, Wastewaters, Soils and Wastes.

[40] (2001). Methods of Testing Soils for Engineering Purposes—Method 1.1: Sampling and Preparation of Soils—Preparation of Disturbed Soil Samples for Testing.

[41] Chubaka C.E., Whiley H., Edwards J.W., Ross K.E. (2018). A Review of Roof Harvested Rainwater in Australia. J. Environ. Public Health.

[42] Iulia M.M., Grace M.V., Richard L.A., Clare D. (2008). Lead and other heavy metals: Common contaminants of rainwater tanks in Melbourne. Water Down Under.

[43] (2021). Australian Government Bureau of Metereology. Climate Data Online. http://www.bom.gov.au/climate/cdo/about/cdo-rainfall-feature.shtml#graphs.

[44] Rayment G.E., Higginson F.R. (1992). Australian Laboratory Handbook of Soil and Water Chemical Methods.

[45] (2009). Methods of Testing Soils for Engineering Purposes—Soil Classification Tests—Determination of the Particle Size Distribution of a Soil—Standard Method of Analysis by Sieving.

[46] Dragomir B., Nicolae C.M. (2017). Soil Characterization in Terms of Granularity and Uniformity. Ann. Univ. Craiova—Agric. Mont. Cadastre Ser..

[47] Khater S. (2023). Development of soil Particle size distribution model and determination of all related coefficients. Ann. Agric. Sci. Moshtohor.

[48] Asakura H., Watanabe Y., Ono Y., Yamada M., Inoue Y., Alfaro A.M. (2010). Characteristics of fine processed construction and demolition waste in Japan and method to obtain fines having low gypsum component and wood contents. Waste Manag. Res..

[49] Lategano E., Costa G., Lombardi F., Baciocchi R. Characterization of the bottom ash produced in a sanitary waste incineration facility and influence of the operating conditions aimed at material recovery or safe disposal. Proceedings of the Sardinia 2007: Eleventh International Waste Management and Landfill Symposium.

[50] Rodrigues F., Carvalho M.T., Evangelista L., De Brito J. (2013). Physical–chemical and mineralogical characterization of fine aggregates from construction and demolition waste recycling plants. J. Clean. Prod..

[51] (1997). Wastes, Sediments and Contaminated Soils. Part 3. Preparation of Leachates—Bottle Leaching Procedure.

[52] (2014). Standard Test Method for Leaching Solid Material in a Column Apparatus.

[53] Somasundaram S., Jeon T.-W., Kang Y.-Y., Kim W.-I., Jeong S.-K., Kim Y.-J., Yeon J.-M., Shin S.K. (2015). Characterization of wastes from construction and demolition sector. Environ. Monit. Assess..

[54] Saca N., Dimache A., Radu L., Iancu I. (2017). Leaching behavior of some demolition wastes. J. Mater. Cycles Waste Manag..

[55] Min H., O’Loughlin E.J., Kwon M.J. (2024). Anaerobic microbial metabolism in soil columns affected by highly alkaline pH: Implication for biogeochemistry near construction and demolition waste disposal sites. J. Environ. Manag..

[56] Sharon B. (2003). Timber leachates prompt preservative review. Eng. Aust..

[57] Deborah R. (2003). Report On Copper, Chromium and Arsenic (CCA) Treated Timber.

[58] Pickin J., Randell P., Trinh J., Grant B., Department of the Environment and Energy (2018). National Waste Report 2018.

[59] Gallen C., Drage D., Kaserzon S., Baduel C., Gallen M., Banks A., Broomhall S., Mueller J.F. (2016). Occurrence and distribution of brominated flame retardants and perfluoroalkyl substances in Australian landfill leachate and biosolids. J. Hazard. Mater..

[60] Kim J. (2021). Construction and demolition waste management in Korea: Recycled aggregate and its application. Clean Technol. Environ. Policy.

[61] Saito T., Kumara G., Matsuno A., Kawamoto K. (2018). Neutralization of acid discharged water around the Kusatsu hot spring area in Japan using construction and demolition wastes. AGU Fall Meeting Abstracts.

[62] Pallewatta S., Weerasooriyagedara M., Bordoloi S., Sarmah A.K., Vithanage M. (2023). Reprocessed construction and demolition waste as an adsorbent: An appraisal. Sci. Total Environ..

[63] Damrongsiri S. (2017). Feasibility of using demolition waste as an alternative heavy metal immobilising agent. J. Environ. Manag..

[64] Santos R.P., Tubino R. (2021). Potential evaluation of the use of construction and demolition waste (CDW) in the recovery of degraded soils by mining in Brazil. Resour. Conserv. Recycl. Adv..

[65] Hyks J., Astrup T., Christensen T.H. (2009). Leaching from MSWI bottom ash: Evaluation of non-equilibrium in column percolation experiments. Waste Manag..

[66] Hyks J., Astrup T., Christensen T.H. (2009). Long-term leaching from MSWI air-pollution-control residues: Leaching characterization and modeling. J. Hazard. Mater..

[67] Dijkstra J.J. (2007). Development of a Consistent Geochemical Modelling Approach for Leaching and Reactive Transport Prosesses in Contaminated Materials. Ph.D. Thesis.

[68] Nakao K., Shakya S., Nozaki T., Inazumi S. (2023). Neutralization Treatment for Recycling Construction-Generated Soils. Appl. Sci..

[69] Sloot H.A.v.d., Zomeren A.v. (2012). Characterisation Leaching Tests and Associated Geochemical Speciation Modelling to Assess Long Term Release Behaviour from Extractive Wastes. Mine Water Environ..

[70] Quina M.J., Bordado J.C., Quinta-Ferreira R.M. (2009). The influence of pH on the leaching behaviour of inorganic components from municipal solid waste APC residues. Waste Manag..

[71] Luo H., Cheng Y., He D., Yang E.-H. (2019). Review of leaching behavior of municipal solid waste incineration (MSWI) ash. Sci. Total Environ..

[72] NSW DECC (2014). Waste Classification Guidelines–Part 1: Classifying Waste. https://www.epa.nsw.gov.au/sites/default/files/140796-classify-waste.pdf.

[73] Australian and New Zealand Environment and Conservation Council, Agriculture and Resource Management Council of Australia and New Zealand (2000). Australian and New Zealand Guidelines for Fresh and Marine Water Quality; Volume 1, pp. 1–314. https://www.waterquality.gov.au/guidelines/anz-fresh-marine.

[74] Gao X., Gu Y., Xie T., Zhen G., Huang S., Zhao Y. (2015). Characterization and environmental risk assessment of heavy metals in construction and demolition wastes from five sources (chemical, metallurgical and light industries, and residential and recycled aggregates). Environ. Sci. Pollut. Res..

[75] Diotti A., Perèz Galvin A., Piccinali A., Plizzari G., Sorlini S. (2020). Chemical and Leaching Behavior of Construction and Demolition Wastes and Recycled Aggregates. Sustainability.

[76] Hyder Consulting, Encycle Consulting, Sustainable Resource Solutions (2011). Hyder Consulting, Encycle Consulting, and Sustainable Resource Solutions, Management of Construction and Demolition Waste in Australia. Construction and Demolition Waste Status Report.

[77] Jang Y.-C., Townsend T.G. (2003). Effect of Waste Depth on Leachate Quality from Laboratory Construction and Demolition Debris Landfills. Environ. Eng. Sci..

[78] Chen Z., Feng Q., Yue R., Chen Z., Moselhi O., Soliman A., Hammad A., An C. (2022). Construction, renovation, and demolition waste in landfill: A review of waste characteristics, environmental impacts, and mitigation measures. Environ. Sci. Pollut. Res..

[79] Daryabeigi Zand A. (2024). Assessing the influence of particle size and dissolved organic carbon on heavy metal leaching from construction and demolition waste modified with carbon-rich materials. Adv. Environ. Technol..

[80] Diotti A., Plizzari G., Sorlini S. (2021). Leaching Behaviour of Construction and Demolition Wastes and Recycled Aggregates: Statistical Analysis Applied to the Release of Contaminants. Appl. Sci..

[81] Zhang J., Kim H., Dubey B., Townsend T. (2017). Arsenic leaching and speciation in C&D debris landfills and the relationship with gypsum drywall content. Waste Manag..

[82] Van Praagh M., Modin H. (2016). Leaching of chloride, sulphate, heavy metals, dissolved organic carbon and phenolic organic pesticides from contaminated concrete. Waste Manag..

[83] Timothy T., Thabet T., Kevin L., Jenna J. (2004). Heavy metals in recovered fines from construction and demolition debris recycling facilities in Florida. Sci. Total Environ..

[84] Mondal T., Choudhury M., Kundu D., Dutta D., Samanta P. (2023). Landfill: An eclectic review on structure, reactions and remediation approach. Waste Manag..

[85] Eckbo C., Okkenhaug G., Hale S.E. (2022). The effects of soil organic matter on leaching of hexavalent chromium from concrete waste: Batch and column experiments. J. Environ. Manag..

[86] Rubinos D.A., Spagnoli G. (2018). Utilization of waste products as alternative landfill liner and cover materials—A critical review. Crit. Rev. Environ. Sci. Technol..

[87] Jong T., Parry D.L. (2003). Removal of sulfate and heavy metals by sulfate reducing bacteria in short-term bench scale upflow anaerobic packed bed reactor runs. Water Res..

[88] Zhuang F., Xiang X., Hu J., Xiong J., Zhang T., Zhou L., Jiang G., Zhang M., Liu Z., Yin H. (2024). Behavior and Mechanisms of Antimony Precipitation from Wastewater by Sulfate-Reducing Bacteria Desulfovibrio desulfuricans. Toxics.

[89] Xie S., Ma Y., Strong P.J., Clarke W.P. (2015). Fluctuation of dissolved heavy metal concentrations in the leachate from anaerobic digestion of municipal solid waste in commercial scale landfill bioreactors: The effect of pH and associated mechanisms. J. Hazard. Mater..

[90] Mahedi M., Cetin B., Dayioglu A.Y. (2019). Leaching behavior of aluminum, copper, iron and zinc from cement activated fly ash and slag stabilized soils. Waste Manag..

[91] Boukerche I., Djerad S., Benmansour L., Tifouti L., Saleh K. (2014). Degradability of aluminum in acidic and alkaline solutions. Corros. Sci..

[92] Cheng W.-P., Fu C.-H., Chen P.-H., Yu R.-F. (2012). Dynamics of aluminum leaching from water purification sludge. J. Hazard. Mater..

[93] Wang L., Cho D.-W., Tsang D.C.W., Cao X., Hou D., Shen Z., Alessi D.S., Ok Y.S., Poon C.S. (2019). Green remediation of As and Pb contaminated soil using cement-free clay-based stabilization/solidification. Environ. Int..

[94] Rodríguez-Jordá M.P., Garrido F., García-González M.T. (2010). Potential use of gypsum and lime rich industrial by-products for induced reduction of Pb, Zn and Ni leachability in an acid soil. J. Hazard. Mater..

[95] Lee S.-W., Lowry G.V., Hsu-Kim H. (2016). Biogeochemical transformations of mercury in solid waste landfills and pathways for release. Environ. Sci. Process. Impacts.

[96] Manceau A., Lemouchi C., Enescu M., Gaillot A.-C., Lanson M., Magnin V., Glatzel P., Poulin B.A., Ryan J.N., Aiken G.R. (2015). Formation of Mercury Sulfide from Hg(II)–Thiolate Complexes in Natural Organic Matter. Environ. Sci. Technol..

[97] Ali H., Khan E., Ilahi I. (2019). Environmental Chemistry and Ecotoxicology of Hazardous Heavy Metals: Environmental Persistence, Toxicity, and Bioaccumulation. J. Chem..

[98] Garai P., Banerjee P., Mondal P., Saha N. (2021). Effect of heavy metals on fishes: Toxicity and bioaccumulation. J. Clin. Toxicol..

[99] Raj D., Maiti S.K. (2020). Sources, bioaccumulation, health risks and remediation of potentially toxic metal(loid)s (As, Cd, Cr, Pb and Hg): An epitomised review. Environ. Monit. Assess..

[100] Ali H., Khan E. (2018). Bioaccumulation of non-essential hazardous heavy metals and metalloids in freshwater fish. Risk to human health. Environ. Chem. Lett..

[101] Zaynab M., Al-Yahyai R., Ameen A., Sharif Y., Ali L., Fatima M., Khan K.A., Li S. (2022). Health and environmental effects of heavy metals. J. King Saud Univ.-Sci..

[102] Wang J., Ma L.Q., Letcher R., Bradford S.A., Feng X., Rinklebe J. (2022). Biogeochemical cycle of mercury and controlling technologies: Publications in critical reviews in environmental science & technology in the period of 2017–2021. Crit. Rev. Environ. Sci. Technol..

[103] Ma M., Hongxia D., Wang D. (2019). Mercury methylation by anaerobic microorganisms: A review. Crit. Rev. Environ. Sci. Technol..

[104] Hellal J., Guédron S., Huguet L., Schäfer J., Laperche V., Joulian C., Lanceleur L., Burnol A., Ghestem J.-P., Garrido F. (2015). Mercury mobilization and speciation linked to bacterial iron oxide and sulfate reduction: A column study to mimic reactive transfer in an anoxic aquifer. J. Contam. Hydrol..

[105] Houlihan M., Bilgen G., Dayioglu A.Y., Aydilek A.H. (2021). Geoenvironmental evaluation of RCA-stabilized dredged marine sediments as embankment material. J. Mater. Civ. Eng..

[106] Robey N.M., Solo-Gabriele H.M., Jones A.S., Marini J., Townsend T.G. (2018). Metals content of recycled construction and demolition wood before and after implementation of best management practices. Environ. Pollut..

[107] Tafu M., Chohji T. (2007). Leaching behaviour of impurities in waste gypsum board. WIT Trans. Ecol. Environ..

[108] Hobson P.N., Bousfield S., Summers R., Kirsch E.J. (1974). Anaerobic digestion of organic matter. C R C Crit. Rev. Environ. Control.

[109] Akfas F., Elghali A., Toubri Y., Samrane K., Munoz M., Bodinier J.-L., Benzaazoua M. (2024). Environmental assessment of phosphogypsum: A comprehensive geochemical modeling and leaching behavior study. J. Environ. Manag..

[110] Liu Y., Molinari S., Dalconi M.C., Valentini L., Ricci G., Carrer C., Ferrari G., Artioli G. (2023). The leaching behaviors of lead, zinc, and sulfate in pyrite ash contaminated soil: Mineralogical assessments and environmental implications. J. Environ. Chem. Eng..

[111] Shruthi, Prakash N.B., Dhumgond P., Goiba P.K., Laxmanarayanan M. (2024). The benefits of gypsum for sustainable management and utilization of acid soils. Plant Soil.

[112] Zhao S., Duan Y., Lu J., Gupta R., Pudasainee D., Liu S., Liu M., Lu J. (2018). Thermal stability, chemical speciation and leaching characteristics of hazardous trace elements in FGD gypsum from coal-fired power plants. Fuel.

[113] Ammar R., El Samrani A.G., Kazpard V., Bassil J., Lartiges B., Saad Z., Chou L. (2013). Applying physicochemical approaches to control phosphogypsum heavy metal releases in aquatic environment. Environ. Sci. Pollut. Res..

[114] Papaslioti E.-M., Pérez-López R., Parviainen A., Sarmiento A.M., Nieto J.M., Marchesi C., Delgado-Huertas A., Garrido C.J. (2018). Effects of seawater mixing on the mobility of trace elements in acid phosphogypsum leachates. Mar. Pollut. Bull..

[115] Deng H., Tian C., Li L., Liang Y., Yan S., Hu M., Xu W., Lin Z., Chai L. (2022). Microinteraction Analysis between Heavy Metals and Coexisting Phases in Heavy Metal Containing Solid Wastes. ACS EST Eng..

[116] Coudray C., Amant V., Cantegrit L., Le Bocq A., Thery F., Denot A., Eisenlohr L. (2017). Influence of Crushing Conditions on Recycled Concrete Aggregates (RCA) Leaching Behaviour. Waste Biomass Valorization.

[117] Nurhanim A., Norli I., Morad N., Khalil H. (2016). Leaching behavior of construction and demolition waste (concrete and gypsum). Iran. J. Energy Environ..

[118] Tayibi H., Choura M., López F.A., Alguacil F.J., López-Delgado A. (2009). Environmental impact and management of phosphogypsum. J. Environ. Manag..

[119] Lee S., Chang H., Lee J. (2024). Construction and demolition waste management and its impacts on the environment and human health: Moving forward sustainability enhancement. Sustain. Cities Soc..

